# Personal Characteristics and Experience of Primary Care Predicting Frequent Use of Emergency Department: A Prospective Cohort Study

**DOI:** 10.1371/journal.pone.0157489

**Published:** 2016-06-14

**Authors:** Catherine Hudon, Steven Sanche, Jeannie L. Haggerty

**Affiliations:** 1 Department of Family Medicine and Emergency Medicine, Université de Sherbrooke, Sherbrooke, Québec, Canada; 2 St Mary’s Research Centre, St Mary’s Hospital, Montréal, Québec, Canada; 3 Department of Family Medicine, McGill University, Montréal, Québec, Canada; National Institute for Viral Disease Control and Prevention, CDC, China, CHINA

## Abstract

**Objective:**

A small number of patients frequently using the emergency department (ED) account for a disproportionate amount of the total ED workload and are considered using this service inappropriately. The aim of this study was to identify prospectively personal characteristics and experience of organizational and relational dimensions of primary care that predict frequent use of ED.

**Methods:**

This study was conducted among parallel cohorts of the general population and primary care patients (N = 1,769). The measures were at baseline (T_1_), 12 (T_2_) and 24 months (T_3_): self-administered questionnaire on current health, health behaviours and primary care experience in the previous year. Use of medical services was confirmed using administrative databases. Mixed effect logistic regression modeling identified characteristics predicting frequent ED utilization.

**Results:**

A higher likelihood of frequent ED utilization was predicted by lower socioeconomic status, higher disease burden, lower perceived organizational accessibility, higher number of reported healthcare coordination problems and not having a complete annual check-up, above and beyond adjustment for all independent variables.

**Conclusions:**

Personal characteristics such as low socioeconomic status and high disease burden as well as experience of organizational dimensions of primary care such as low accessibility, high healthcare coordination problems and low comprehensiveness of care are prospectively associated with frequent ED utilization. Interventions developed to prevent inappropriate ED visits, such as case management for example, should tailor low socioeconomic status and patients with high disease burden and should aim to improve experience of primary care regarding accessibility, coordination and comprehensiveness.

## Introduction

Patients who make frequent use of the emergency department (ED) account for a disproportionate amount of the total ED workload[1,2].The definition of frequent use varies, ranging from three to twelve ED visits within a year, but most authors define frequent users as those making at least three or four ED visits in a 12-month period [1,3–7]. These frequent users are often viewed as inappropriately using the ED for nonmedical or non-urgent reasons [2,5,8,9]. They often suffer from a complex array of psychosocial problems, which might compound chronic medical conditions [7,10] and they also more often tend to be substance abusers (including alcohol) [4,9,11]. This population has complex needs[12,13] and is at higher risk of hospital admission[2] and premature death[5,10].

Frequent ED visits may also be a symptom of primary care failures in continuity, accessibility or comprehensiveness [14–16]. Complications of certain chronic medical conditions (e.g., diabetes, asthma, congestive heart failure) are considered to be particularly sensitive to access to primary care services [17]. Better primary care management of these conditions may improve the process of care and clinical outcomes [18–20] and reduce the probability of complications or clinical deterioration that could lead to an ED visit or hospitalization [21]. The delivery of a complete annual check-up is thought to be linked to comprehensiveness of care[22], and was associated with a lower ED utilization in a retrospective cohort of adults[23]. Even if research about the impact of relational aspects of primary care on ED utilization is sparse, studies have documented that empowerment or patient centered care could decrease healthcare utilization [24,25].

Although literature suggests that some personal [2,4,6,9,11,26] and primary care characteristics [14–16] could be associated with ED utilization, few studies examined these variables in the same model and in a prospective design. The aim of this study was to identify prospectively personal characteristics and experience of organizational and relational dimensions of primary care that predict frequent use of ED.

## Methods

The present study is an analysis conducted among parallel cohorts of the general population and of primary care patients recruited in the context of a primary care cohort study (the Program of Research on the Evolution of a Cohort Investigating Health System Effects, PRECISE)[27] within the geographic boundaries of four local healthcare networks in Québec, Canada. These networks are located in metropolitan, urban, rural and remote settings. The study was approved by the Ethics Committees of the Centre de santé et de services sociaux de Chicoutimi and of Hôpital Charles Lemoyne. Informed written consent was obtained from every participant. Data was de-identified prior to analysis. Only the alpha-numeric identity code was used, and the link to nominal information was not accessible to the analyst.

### Selection of participants

A population cohort was recruited from March to April 2010 through a telephone survey by random digit dialing of telephone numbers mapped to the postal code areas that correspond to the administrative boundaries of the four networks identified. Once contact was made, staff selected the adult in the household whose birthday was closest to the date of the interview to ensure random selection[28].

A second clinical cohort was also recruited from March to April 2010 from patients in the waiting room of 12 primary care clinics. In each of the four networks, we purposefully selected three sentinel clinics typical of the dominant forms of primary healthcare organizations: private medical clinics, community health clinics, and Family Medicine Groups. To be included in the study, participants had to be regular patients of the clinic or be consulting for themselves. All participants had to be aged between 25 and 75 years, able to respond to written and oral questions in English or French and reside in one of the four identified networks.

### Study design and setting

At baseline (T_1_), cohort participants were mailed (population cohort) or given (clinical cohort) a self-administered questionnaire on their sociodemographic information, current health, health behaviors and primary healthcare experience in the previous year. The questionnaire was mailed again at 12 months (T_2_) and 24 months (T_3_). Consent was requested to access their record of claims for medical services from the Quebec healthcare agency (RAMQ).

### Methods and Measurements

**T**_**1**_**, T**_**2**_**, and T**_**3**_—The self-administered questionnaire containing approximately 160 questions was available in paper format (mailed) or online. Participants with chronic diseases responded to an additional set of 32 questions.

We applied the Dillman method [29] to maximize response to questionnaires at T_1_, T_2_ and T_3_. Compensation was mailed with the questionnaire to enhance response[30]. Subjects were considered lost-to-follow-up after eight weeks of non-response or explicit refusal to continue to participate.

#### Independent variables (personal and primary care characteristics)

Personal characteristics included sociodemographic and socioeconomic variables as well as health behaviors and health status variables. Sociodemographic and socioeconomic variables are based on cluster analysis. We identified respondents as having low **socioeconomic status** if they reported all of the following conditions: 1) lower educational attainment (high school diploma or less); 2) very poor, tight or modest self-perceived financial situation and 3) neither having personal retirement fund nor private medication insurance.

**Urban/rural region** was determined with postal code. The postal code of participants was associated to the type of regions using software created from the national statistical office. Urban regions consist of one or multiple municipalities located near urban cores (regions having a density of at least 400 inhabitants per square km with a total population above 10,000). Nearby municipalities are annexed if at least 50% of the active population commute to the core. All other regions are qualified as rural.

We measured **illness burden** using the validated Disease Burden Morbidity Assessment[31,32], where for each of 22 physical and mental conditions diagnosed by a health professional, the person reports the extent to which the illness interferes with daily activities on a scale of 1 to 5. **Mental health status** was measured using the mental component of SF-12v2[33]. We measured **psychological distress** using the K6[34], with a score of ≥10 indicating high risk of anxiety or depression. We measured the presence and intensity of **alcohol consumption** using validated sub-scales from the Behavior Risk Factor Surveillance System Questionnaire[35], but we applied the widely publicized Educalcool cut-off for high risk alcohol consumption (more than 10 standard drinks per week for women; more than 15 standard drinks per week for men) [36]. **Self-efficacy** was evaluated with a one-question (5 levels) about patient self-perception of control over his/her health.

Primary care characteristics were grouped into two categories: **organizational and relational characteristics**. To measure **organizational characteristics,** each respondent was asked to report on their experience over the previous 12 months with his/her usual primary care clinic using validated subscales: **organizational accommodation and availability**[37]; and **number of coordination problems** encountered[38]. **Complete annual check-ups** were identified from the claims database from procedure codes for a complete annual check-up, which consists in an examination of three or more physiologic systems that can only be performed once per patient per year by a family physician.

**Interpersonal communication** with the regular family physician was evaluated with the PCAS communication scale (6 items) [39]. **Empowerment** was evaluated with the 3-items sub-scale of the interpersonal processes of care[40].

#### Dependent variable

We used claims database to ascertain all ED visits. Because there could be more than one ED billing per visit, we identified single visits using all billings on up to two consecutive days[41]. We measured the number of ED visits per patient per year for the 2 years immediately following the completion of T_1_ and T_2_ questionnaires. Frequent users were defined operationally as those who made 3 or more ED visits during the year.

### Analysis

**Fig 1:**
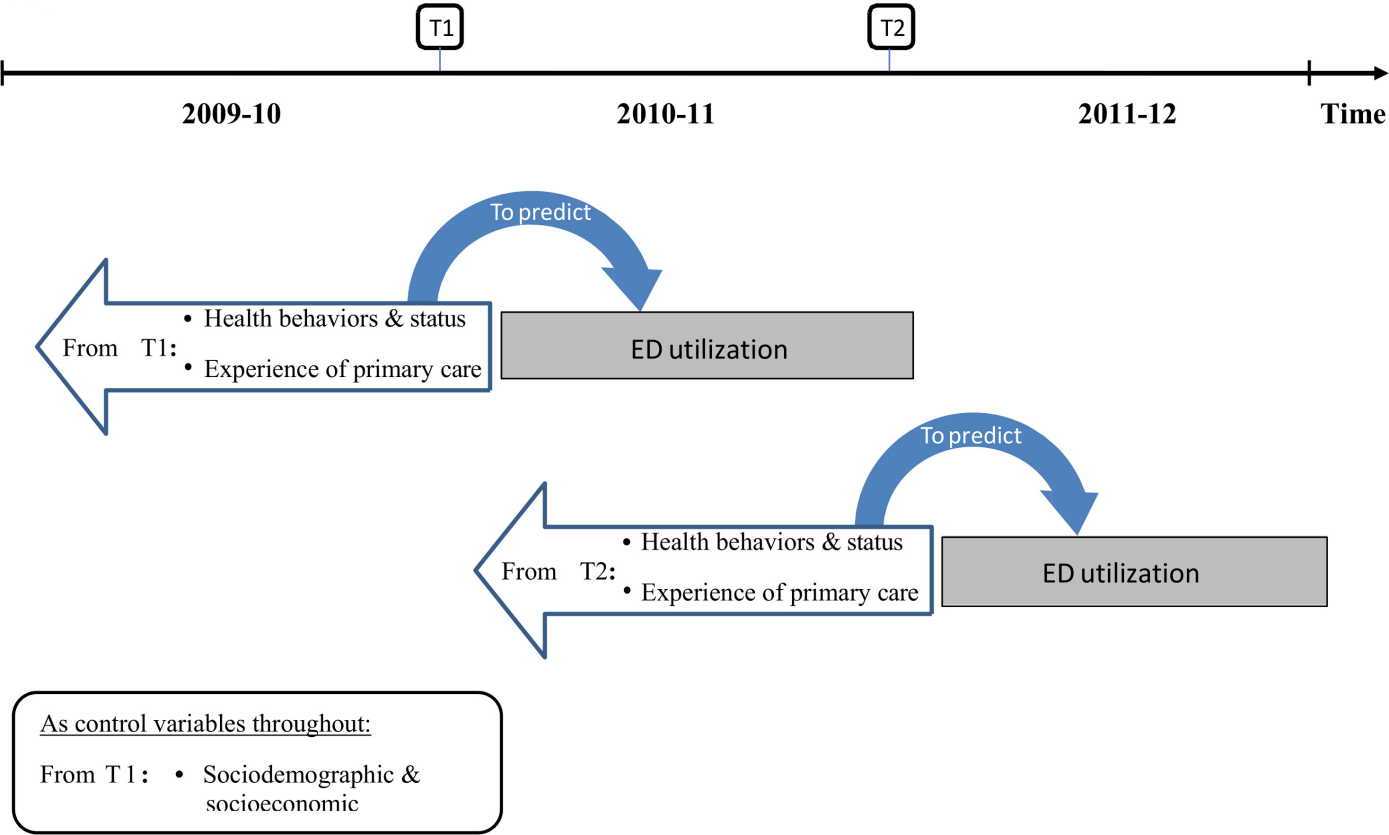
Mixed effects logistic regression modeling for the study.

General descriptive statistics were obtained for all independent and dependent variables. Mixed effects logistic regression modeling was performed to identify the characteristics associated with a higher likelihood of being a frequent ED user during the subsequent year (Fig 1). All regression models simultaneously included the two outcome measurements: frequent ED use on the year following T_1_ and frequent ED use on the year following T_2_. We modeled the impact of predictors measured at T1 on the outcome measured in 2010–11. Similarly, we modeled the impact of predictors measured at T2 on the outcome measured in 2011–12. Covariate values were only measured once at T1. The model assumed that the relationship between predictor and ED use is similar for both years (we fitted one parameter for both). The plausibility of this assumption was investigated by fitting data years separately.

Random effects were used to account for the lack of independence between the outcome values, which could emanate from the repetition of the ED utilization measure at two time points or the sampling design. Indeed, patients were selected within a sample of clinics, in the clinical cohort, and this could result in correlated outcome values between patients of the same clinics. We accounted for the lack of independence between two such patients by using a random intercept for clinics in the regression models. We further accounted for the lack of independence between outcomes measured at two time points on the same patient, by allowing the corresponding residuals to covary. Finally, we considered the potential difference in ED utilization between the population and the clinical cohort by allowing the former to have a different intercept. All measures were obtained at the patient level. Fixed effects were used to model the impact of all covariates (sociodemographic or socioeconomic variables) and all predictors (health behaviors and status, primary care organizational and relational characteristics).

Multiple models were fitted, using as independent variable(s): 1) one predictor or covariate at a time; 2) the set of personal characteristics (sociodemographic, socioeconomic, health behaviors and status), 3) the set of healthcare experience measures (organizational and relational) and 4) all independent variables in a single model.

Power analysis was performed using numerical simulation of the outcome based on a model observing the same covariance structure as realized in the data. We simulated the effect of continuous and dichotomous predictors. The continuous predictor followed a normal distribution, and the dichotomous predictor had a prevalence of 10 or 25%.

Variance inflation estimates were obtained. No variable selection procedure based on p-values was used; these procedures produce biased effect and significance estimates[42]. Using approximate regression rules[42], we estimated that the number of events (frequent ED users) allowed for the modeling of around 15 independent variables without over-fitting the data (10 events per parameter).

There were no missing values for any of the studied dependent variables. Overall, 15% of the cases contained missing values for one or more of the independent variables. Analyses were performed using SAS’ GLIMMIX procedure, which eliminates complete-case bias by incorporating all available information[43]. All statistical analyses were performed using SAS 9.3 (SAS Institute, Cary NC). A 95% confidence level was used in all analyses.

## Results

### Characteristics of study subjects

**Fig 2:**
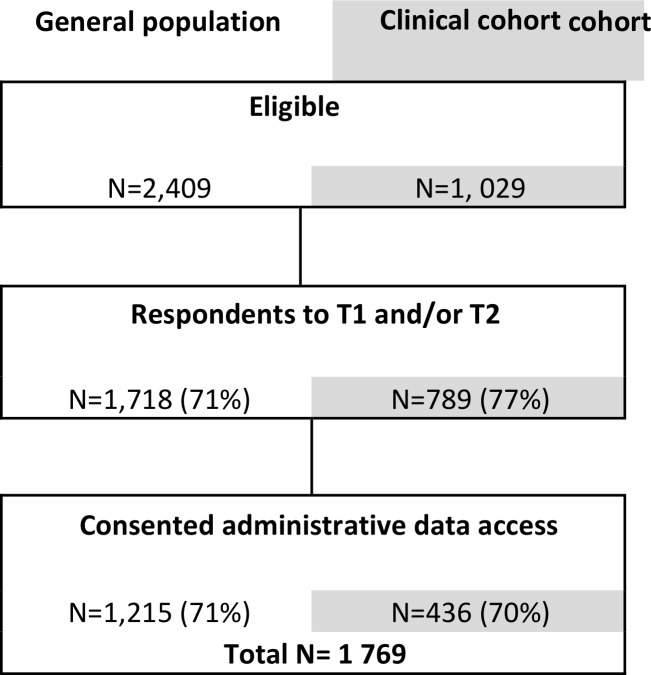
Sample flow chart: the sample size at sampling steps and the percentage kept from the previous step.

The sample that was used for analyses consisted of the participants who responded to at least one of the questionnaires T1 and T2, and which data could be linked to administrative databases (N = 1,769). The eligible population from the general population cohort and the clinical cohort was composed of 2 409 and 1 029 participants respectively, from which 71% and 77% responded to one or two questionnaires. The percentage of participants consenting access to their administrative data was about 70% (Fig 2). A notable percentage of the analysis sample (88%) responded to both questionnaires.

Tables 1–3 report descriptive statistics for all independent variables. Each 10-year age bracket, starting from 25 years, was represented by at least 190 participants. The average age over the sample was 53 years old. Fifty-nine (59%) percent of the sample was female. The urban/rural residency context was well proportioned, with 45% of the sample living in a rural area. Fourteen percent of the sample did not possess a high school diploma and considered themselves to be in a financially tight situation.

**Table 1:** Sociodemographic data of both cohorts.

Variables			Population cohort		Clinical cohort		Total	
			N	%	N	%	N	%
**Sociodemographic and socioeconomic variables**								
	**Age**							
		25–34	142	11.7	50	10.1	192	11.3
		35–44	191	15.8	71	14.4	262	15.4
		45–54	312	25.7	123	24.9	435	25.5
		55–64	345	28.4	133	26.9	478	28
		65–74	211	17.4	108	21.9	319	18.7
		75+ (max = 77)	12	1	9	1.8	21	1.2
		Missing	2		60		62	
	**Sex**							
		Male	520	42.9	175	35	695	40.6
		Female	693	57.1	325	65	1018	59.4
		Missing	2		54		56	
	**Socioeconomic status**							
		Low socioeconomic status	153	13.1	73	15.7	226	13.9
		Moderate or high socioeconomic status	1015	86.9	391	84.3	1406	86.2
		Missing	47		90		137	
	**Area of residence**							
		Urban	592	49.5	330	68.6	922	55
		Rural	604	50.5	151	31.4	755	45
		Missing	19		73		92	

**Table 2:** Personal characteristics about health behaviors and status.

Variables	T1						T2					
	Population cohort		Clinical cohort		Total		Population cohort		Clinical cohort		Total	
	N	%	N	%	N	%	N	%	N	%	N	%
**Health behaviors and status**												
** Disease Burden score**												
0	389	32.2	131	27.1	520	30.7	299	27.0	99	23.1	398	25.9
1–2	336	27.8	129	26.7	465	27.5	332	29.9	113	26.3	445	28.9
3–4	224	18.5	76	15.7	300	17.7	201	18.1	84	19.6	285	18.5
5–10	206	17.1	101	20.9	307	18.1	220	19.8	93	21.7	313	20.4
11–15	53	4.4	47	9.7	100	5.9	57	5.1	40	9.3	97	6.3
Missing	7		70		77		106		125		231	
** Mental health functioning score**												
10–29 (min = 12)	37	3.1	28	5.9	65	3.9	30	2.7	26	6.3	56	3.7
30–39	145	12.2	67	14.1	212	12.8	111	10.2	49	11.8	160	10.6
40–49	288	24.3	138	29.1	426	25.7	260	23.8	107	25.8	367	24.4
50–59	527	44.5	177	37.3	704	42.4	533	48.9	180	43.4	713	47.3
60+ (max = 72)	187	15.8	65	13.7	252	15.2	157	14.4	53	12.8	210	13.9
Missing	31		79		110		124		139		263	
** At risk for psychological distress**												
No	1143	94.9	435	89.3	1578	93.3	1066	95.9	398	93.0	1464	95.1
Yes	62	5.1	52	10.7	114	6.7	45	4.1	30	7.0	75	4.9
Missing	10		67		77		104		126		230	
** Alcohol consumption**												
High	203	17.3	68	14.4	271	16.5	158	14.4	51	12.1	209	13.7
None, low or moderate	971	82.7	403	85.6	1374	83.5	941	85.6	372	87.9	1313	86.3
Missing	41		83		124		116		131		247	
** Self-efficacy**												
1	26	2.2	18	3.7	44	2.7	10	0.9	11	2.6	21	1.4
2	51	4.3	27	5.6	78	4.7	53	4.8	26	6.1	79	5.1
3	147	12.5	56	11.6	203	12.2	99	8.9	48	11.2	147	9.6
4	554	47.0	229	47.6	783	47.2	494	44.5	193	45.2	687	44.7
5	401	34.0	151	31.4	552	33.3	454	40.9	149	34.9	603	39.2
Missing	36		73		109		105		127		232	

**Table 3:** Organizational and relational characteristics.

Variables	T1						T2					
	Population cohort		Clinical cohort		Total		Population cohort		Clinical cohort		Total	
	N	%	N	%	N	%	N	%	N	%	N	%
**Health care experience**												
**Organizational characteristics**												
** Has a family physician**												
No	117	9.7	32	6.7	149	8.8	only measured at T1					
Yes	1090	90.3	449	93.3	1539	91.2						
Missing	8		73		81							
** Organizational accessibility score**												
[1;2[	110	9.1	42	8.6	152	9.0	115	10.4	43	9.9	158	10.3
[2;3[	368	30.6	154	31.7	522	30.9	284	25.7	121	27.8	405	26.3
[3;4[	471	39.1	185	38.1	656	38.8	395	35.8	140	32.2	535	34.8
[4;5]	255	21.2	105	21.6	360	21.3	309	28.0	131	30.1	440	28.6
Missing	11		68		79		112		119		231	
** Coordination issues encountered**												
0	465	40.2	168	35.4	633	38.8	477	43.8	181	42.2	658	43.3
1	200	17.3	95	20.0	295	18.1	183	16.8	67	15.6	250	16.5
2	178	15.4	68	14.3	246	15.1	152	13.9	50	11.7	202	13.3
3	120	10.4	55	11.6	175	10.7	105	9.6	42	9.8	147	9.7
4+	195	16.8	88	18.6	283	17.3	173	15.9	89	20.7	262	17.2
Missing	57		80		137		125		125		250	
** Had a complete annual check-up**												
No	847	69.7	355	64.1	1202	67.9	823	67.7	401	72.4	1224	69.2
Yes	368	30.3	199	35.9	567	32.1	392	32.3	153	27.6	545	30.8
Missing	0		0									
**Relational characteristics**												
** Communication score**												
[1;3[	42	3.5	16	3.3	58	3.5	50	4.6	19	4.4	69	4.6
[3;4[	137	11.5	62	12.8	199	11.9	136	12.5	57	13.3	193	12.7
[4;5[	368	30.8	134	27.7	502	29.9	312	28.7	119	27.7	431	28.4
[5;6]	648	54.2	271	56.1	919	54.8	588	54.1	234	54.5	822	54.3
Missing	20		71		91		129		125		254	
** Empowerment score**												
[1;2[	141	12.0	47	9.8	188	11.3	59	5.4	29	6.8	88	5.8
[2;3[	160	13.6	66	13.7	226	13.6	126	11.6	43	10.0	169	11.2
[3;4[	330	28.1	125	25.9	455	27.5	306	28.3	123	28.7	429	28.4
[4;5]	544	46.3	244	50.6	788	47.6	592	54.7	233	54.4	825	54.6
Missing	40		72		112		132		126		258	

In terms of health behaviors and status, most individuals reported experiencing few limitations (55 to 58% of the sample had a DBMA score of less than 2), but those who reported otherwise experienced a wide range of limitations (from 3 to 15). Similarly, most individuals had an average mental health functioning score, but those who had not had a wide range of scores (the lowest being a score of 12). However, few individuals were considered at risk of psychological distress (approximately 5%). Those who were considered having high alcohol consumption were better represented (14 to 17%). A relatively small number of individuals had a low self-efficacy score (7 to 9% had a score of 2 or less).

A notable range of health experiences was reported. Most individuals (91%) reported having a family physician. Furthermore, most individuals reported having encountered at least 1 coordination issue, with around 17% having encountered 4 or more. In terms of organizational accessibility and empowerment, most individuals obtained a high score (better score), with 9 to 12% of the sample having less than 2 on the score scale ranging from 1 to 5. Less variation was observed for the communication scale, with only 3.5 to 4.5% being in the lowest half part of the scale.

Of all the 1,769 participants, 97 (4.9%) had made at least 3 ED visits during the year following T_1_; 69 (3.9%) for T_2_.

### Power analysis results

The power analysis revealed that we had at least 80% power to detect small effects from continuous variables (odds ratio of 0.77 or inversely 1.30 for an increase of 1 standard deviation), medium effects from dichotomous variables with a prevalence of 25% or more (odds ratio of 0.61 or inversely 1.65), and large effects from dichotomous variables with a prevalence of 10% (odds ratio of 0.5 or inversely 2). The power to detect effects with lower than the above-mentioned effect sizes rapidly decreased.

### Main results

Mixed model regression modeling results are shown in Table 4. The following predicted a higher likelihood of frequent ED utilization above and beyond adjustment for all independent variables: 1) lowest socioeconomic status group; 2) higher disease burden score; 3) lower organizational accommodation and availability score; 4) higher number of reported healthcare coordination problems; 5) no complete annual check-up received. Low socioeconomic status, low organizational accessibility, and lack of a complete annual checkup have a medium to high effect size. Count of coordination issues and high disease burden are significant, but the effect size is small. While being significant in the univariate model, the odds ratio for psychological distress risk is approximately halved after adding both the disease burden score (point-serial correlation of 0.25) and coordination issues (point-biserial correlation of 0.19), both variables having an equal impact on the univariate odds ratio estimate. The small prevalence of people with a high risk of psychological distress made it improbable to detect its effect unless it was very large. The impact of interpersonal communication was essentially altered by the inclusion of the organizational accessibility and coordination issues variables (Pearson correlation of 0.43 and -0.25 respectively). It is possible that the lack of variation of this scale diminished the power to detect its effect beyond medium effects. The impact of the self-efficacy score was sensitive to many of the independent variables. This could be due to lower variability. All variance inflation estimates had lower than 2 values.

**Table 4:** Mixed model regression results for all studied univariate and multivariate models: odds ratio of frequent ED use with 95% confidence intervals.

Variables	Univariate Models	Multivariate model 1: Individual characteristic	Multivariate model 2: Experience characteristics	Final multivariate model
**Confounding variables**				
** **Age	NS	NS	—	NS
** **Female gender	NS	NS	—	NS
** **Lowest SES cluster	2.66 [1.77;3.99]	2.16 [1.38;3.4]	—	1.9 [1.19;3.03]
** **Rurality	0.66 [0.45;0.97]	0.58 [0.38;0.89]	—	NS
**Intrinsic Patient Factors**				
** **Illness burden	1.11 [1.07;1.15]	1.1 [1.05;1.15]	—	1.1 [1.04;1.15]
** **Mental health functioning	0.97 [0.96;0.99]	NS	—	NS
** **High risk psychological distress	3.12 [1.91;5.11]	NS	—	NS
** **High risk alcohol consumption	NS	NS	—	NS
** **Self-efficacy	0.67 [0.57;0.79]	NS	0.73 [0.62;0.87]	NS
**Primary Care Experience**				
** **Organisational accessibility	0.61 [0.51;0.75]	—	0.69 [0.55;0.86]	0.61 [0.48;0.77]
** **Number of coordination issues	1.33 [1.22;1.45]	—	1.24 [1.12;1.36]	1.2 [1.08;1.33]
** **Complete annual checkup	0.56 [0.37;0.83]	—	0.53 [0.34;0.83]	0.6 [0.37;0.95]
** **Interpersonal communication	0.78 [0.66;0.93]	—	NS	NS
** **Empowerment	NS	—	NS	NS
** **Having a family physician	0.59 [0.35;0.99]	—	NS	NS

NS: included in the model, not statistically significant

—: not included in the model

## Discussion

Our results document that personal characteristics such as low socioeconomic status and high disease burden as well as experience of organizational dimensions of primary care such as low organization accessibility, high healthcare coordination problems and low comprehensiveness of care are prospectively associated with frequent ED utilization.

Our study extends previous research on characteristics associated with ED utilization by confirming these associations in the same model. The study used a prospective design, therefore we expect the measures of association between predictors and outcome to be more accurate as compared to similar retrospective cohort studies, where self-reported measures are more subject to recall bias and more likely to be inversely influenced by the occurrence of the outcome (e.g. patients reporting more negatively on the health care system due to frequent ED visits). It is also one of the first prospective studies to explore if the relational dimensions of primary care could predict frequent ED use.

At an individual level, poverty has been often stressed as being associated with frequent ED use [6,10].Our results document that low socioeconomic status has a big impact on the likelihood of making frequent ED visits. The association remained strong even after controlling for potential confounders such as mental health issues or rurality. It is possible that these individuals require more guidance in navigating through the primary healthcare system.

Another important personal characteristic is the reported illness burden, which has a moderate impact on frequent ED use (OR of 1.5 for an increase of one standard deviation of the predictor). This measure is different from traditional chronic disease counts; an individual feeling highly limited by its unique chronic condition could score higher on this scale than one suffering from multiple conditions.

As mentioned above, it has been frequently reported that mental health issues are tightly linked with frequent ED use. After controlling for other factors, the two mental health measures were not significantly linked with frequent ED use. Although there was enough statistical power to detect the effect of the continuous measure of mental health functioning, we could only detect a very large effect of psychological distress. This informs on the unlikelihood of a continuous relationship between mental health functioning and frequent ED use. However, there could still be a threshold impact of mental health where only people with severe mental health issues have frequent ED use. In fact, our results inform us that although the effect of psychological distress is very large without controlling for disease burden and coordination issues, it likely becomes less important (none, small or moderate effect) after controlling for these confounding variables. This suggests that improving the coordination of care and addressing the disease burden issues could partly alleviate ED use in this population.

At an organizational level, previous studies reported that individuals who perceived accessibility and continuity problems in regular care are more likely to utilize the ED for primary care related reasons [14–16,44]. Our results confirm that the experience of accessibility and continuity problems increase frequent use of ED. In fact, the patients’ perceptions of those two main components of care did have a large impact on frequent ED use. Family physicians’ complete annual check-up also improves the delivery of prevention services and may reduce worry in patients,^21-^[45] thus also potentially reducing ED visits. This is in accordance with our results, where a complete annual checkup greatly reduced the likelihood of frequent ED use after controlling for family physician affiliation. It is still unclear at this point whether it is the act of providing comprehensive care which impacts the likelihood of frequent ED use or if it is an underlying factor, such as the inherent propensity of individuals getting complete annual checkup toward prevention.

At a relational level, lower interpersonal communication predicted a higher likelihood of frequent ED utilization but not after controlling for accessibility and coordination issues; the impact of lower interpersonal communication skills is likely relatively small. These are first results regarding relational aspects of primary care in a prospective design. Effects of communication, empowerment and patient-centered care and interaction between organizational and relational attributes of primary care would deserve more attention in further studies on ED utilization.

### Strengths and limitations

Using a prospective design allowed us to verify the temporality of the studied characteristics. We also put special emphasis on the rigor of statistical analyses. Finally, we believe that studying both personal characteristics and experience of primary care provides a better overview of risk factors for greater use of services and allows planning interventions at different levels.

Our study’s limitations are primarily related to the fact that we paired two cohorts from two different populations. However, this aspect was considered in the analysis and revealed no major differences (see S1 Appendix). We defined frequent use as 3 or more ED visits. It would be interesting to see which factors predict very frequent users of ED (e.g., 10 or more visits) and whether this group is particularly sensitive to patient centered care. The prevalence of frequent ED use was low in our sample (4 to 5%), which limited the probability of detecting small to moderate effect sizes for the dichotomous predictors. Finally, the results obtained from developed countries may not hold true for developing countries since healthcare systems are different.

## Conclusion

In summary, personal characteristics such as low socioeconomic status and high disease burden as well as experience of organizational dimensions of primary care such as low organization accessibility, high healthcare coordination problems and low comprehensiveness of care are prospectively associated with frequent ED utilization. Interventions or programs developed to prevent inappropriate ED visits, such as case management for example, should tailor low socioeconomic status and patients with high disease burden and should aim to improve experience of primary care regarding accessibility, coordination and comprehensiveness.

## Supporting Information

S1 Appendix Plausibility of the assumption that the relationship between predictor and ED use was similar for both years. (DOCX)

## Abbreviations

ED: Emergency department

## References

[1] Locker TE, Baston S, Mason SM, Nicholl J. Defining frequent use of an urban emergency department. Emergency medicine journal: EMJ. 2007;24(6):398–401. Epub 2007/05/22. 24/6/398 [pii] 10.1136/emj.2006.043844 17513534 PMC2658272

[2] Miller JB, Brauer E, Rao H, Wickenheiser K, Dev S, Omino R, et al The most frequent ED patients carry insurance and a significant burden of disease. Am J Emerg Med. 2013;31(1):16–9. Epub 2012/07/17. S0735-6757(12)00205-7 [pii] 10.1016/j.ajem.2012.05.001 . 22795986

[3] Zuckerman S, Shen YC. Characteristics of occasional and frequent emergency department users: do insurance coverage and access to care matter? Medical care. 2004;42(2):176–82. Epub 2004/01/22. 10.1097/01.mlr.0000108747.51198.41 . 14734955

[4] Mandelberg JH, Kuhn RE, Kohn MA. Epidemiologic analysis of an urban, public emergency department's frequent users. Academic emergency medicine: official journal of the Society for Academic Emergency Medicine. 2000;7(6):637–46. Epub 2000/07/25. . 10905642 10.1111/j.1553-2712.2000.tb02037.x

[5] Hansagi H, Olsson M, Sjoberg S, Tomson Y, Goransson S. Frequent use of the hospital emergency department is indicative of high use of other health care services. Ann Emerg Med. 2001;37:561–7. 11385324 10.1067/mem.2001.111762

[6] Hunt KA, Weber EJ, Showstack JA, Colby DC, Callaham ML. Characteristics of frequent users of emergency departments. Ann Emerg Med. 2006;48(1):1–8. Epub 2006/06/20. S0196-0644(06)00068-0 [pii] 10.1016/j.annemergmed.2005.12.030 . 16781914

[7] Bieler G, Paroz S, Faouzi M, Trueb L, Vaucher P, Althaus F, et al Social and medical vulnerability factors of emergency department frequent users in a universal health insurance system. Acad Emerg Med. 2012;19(1):63–8. Epub 2012/01/10. 10.1111/j.1553-2712.2011.01246.x . 22221292

[8] Carret ML, Fassa AG, Kawachi I. Demand for emergency health service: factors associated with inappropriate use. BMC Health Serv Res. 2007;7:131 Epub 2007/08/21. 1472-6963-7-131 [pii] 10.1186/1472-6963-7-131 17705873 PMC2034385

[9] Huang JA, Tsai WC, Chen YC, Hu WH, Yang DY. Factors associated with frequent use of emergency services in a medical center. J Formos Med Assoc. 2003;102(4):222–8. Epub 2003/07/02. . 12833184

[10] Chan BT, Ovens HJ. Frequent users of emergency departments. Do they also use family physicians' services? Can Fam Physician. 2002;48:1654–60. Epub 2002/11/27. 12449550 PMC2213944

[11] Cherpitel CJ, Ye Y. Drug use and problem drinking associated with primary care and emergency room utilization in the US general population: data from the 2005 national alcohol survey. Drug Alcohol Depend. 2008;97(3):226–30. 10.1016/j.drugalcdep.2008.03.033 18499355 PMC3007592

[12] Olsson M, Hansagi H. Repeated use of the emergency department: qualitative study of the patient’s perspective. Emergency medicine journal: EMJ. 2001;18:430–4. 11696488 10.1136/emj.18.6.430 PMC1725740

[13] Sun BC, Burstin HR, Brennan TA. Predictors and outcomes of frequent emergency department users. Acad Emerg Med. 2003;10:320–8. 12670845 10.1111/j.1553-2712.2003.tb01344.x

[14] McCusker J, Roberge D, Ciampi A, Levesque JF, Pineault R, Belzile E, et al Primary Care Organization and Outcomes of an Emergency Visit among Seniors. Healthc Policy. 2009;5(1):e115–31. Epub 2010/08/03. 20676243 PMC2732659

[15] Lowe RA, Localio AR, Schwarz DF, Williams S, Tuton LW, Maroney S, et al Association between primary care practice characteristics and emergency department use in a medicaid managed care organization. Medical care. 2005;43(8):792–800. Epub 2005/07/22. 00005650-200508000-00007 [pii]. . 16034293 10.1097/01.mlr.0000170413.60054.54

[16] Haggerty JL, Roberge D, Pineault R, Larouche D, Touati N. Features of primary healthcare clinics associated with patients' utilization of emergency rooms: urban-rural differences. Healthc Policy. 2007;3(2):72–85. Epub 2007/11/01. 19305782 PMC2645171

[17] Brown AD, Goldacre MJ, Hicks N, Rourke JT, McMurtry RY, Brown JD, et al Hospitalization for ambulatory care-sensitive conditions: a method for comparative access and quality studies using routinely collected statistics. Can J Public Health. 2001;92(2):155–9. Epub 2001/05/08. . 11338156 10.1007/BF03404951 PMC6979584

[18] Weingarten SR, Henning JM, Badamgarav E, Knight K, Hasselblad V, Gano A Jr., et al Interventions used in disease management programmes for patients with chronic illness-which ones work? Meta-analysis of published reports. BMJ (Clinical research ed). 2002;325:925 . 12399340 10.1136/bmj.325.7370.925 PMC130055

[19] Ofman JJ, Badamgarav E, Henning JM, Knight K, Gano AD Jr., Levan RK, et al Does disease management improve clinical and economic outcomes in patients with chronic diseases? A systematic review. Am J Med. 2004;117(3):182–92. Epub 2004/08/11. . 15300966 10.1016/j.amjmed.2004.03.018

[20] Tsai AC, Morton SC, Mangione CM, Keeler EB. A meta-analysis of interventions to improve care for chronic illnesses. Am J Manag Care. 2005;11:478–88. 16095434 PMC3244301

[21] McCusker J, Roberge D, Levesque JF, Ciampi A, Vadeboncoeur A, Larouche D, et al Emergency department visits and primary care among adults with chronic conditions. Medical care. 2010;48(11):972–80. Epub 2010/09/22. 10.1097/MLR.0b013e3181eaf86d . 20856143

[22] Da Silva RB, Contandriopoulos A-P, Pineault R, Tousignant P. A Global Approach to Evaluation of Health Services Utilization: Concepts and Measures. Healthcare Policy. 2011;6(4):e106–e17. PMC3107120. 22548101 PMC3107120

[23] McCusker J, Tousignant P, Borges Da Silva R, Ciampi A, Levesque JF, Vadeboncoeur A, et al Factors predicting patient use of the emergency department: a retrospective cohort study. CMAJ. 2012;184(6):E307–16. 10.1503/cmaj.111069 22353588 PMC3314059

[24] Wong CK, Wong WC, Lam CL, Wan YF, Wong WH, Chung KL, et al Effects of Patient Empowerment Programme (PEP) on clinical outcomes and health service utilization in type 2 diabetes mellitus in primary care: an observational matched cohort study. PLoS One. 2014;9(5):e95328 10.1371/journal.pone.0095328 24788804 PMC4006782

[25] Bertakis KD, Azari R. Patient-centered care is associated with decreased health care utilization. J Am Board Fam Med. 2011;24(3):229–39. 10.3122/jabfm.2011.03.100170 . 21551394

[26] Mehl-Madrona LE. Prevalence of psychiatric diagnoses among frequent users of rural emergency medical services. Can J Rural Med. 2008;13(1):22–30. Epub 2008/01/23. . 18208649

[27] Haggerty J, Fortin M, Beaulieu M-D, Hudon C, Loignon C, Préville M, et al At the interface of community and healthcare systems: a longitudinal cohort study on evolving health and the impact of primary healthcare from the patient's perspective. BMC Health Services Research 2010;10:258 10.1186/1472-6963-10-258 20815880 PMC2940881

[28] Bryant H, Robson PJ, Ullman R, Friedenreich C, Dawe U. Population-based cohort development in Alberta, Canada: a feasibility study. Chronic diseases in Canada. 2006;27:51–9. 16867239

[29] Dillman DA. Mail and Telephone Surveys: The Total Design Method New York: John Wiley and Sons; 1978.

[30] Dillman DA. Mail and Internet Surveys The tailored design method. 2nd ed. New York: John Wiley & Sons, Inc.; 2000.

[31] Bayliss EA, Ellis JL, Steiner JF. Subjective assessments of comorbidity correlate with quality of life health outcomes: Initial validation of a comorbidity assessment instrument. Health and Quality of life Outcomes. 2005;3:51 16137329 10.1186/1477-7525-3-51 PMC1208932

[32] Poitras M-E, Fortin M, Hudon C, Haggerty J, Almirall J. Validation of the disease burden morbidity assessment by self-report in a French-speaking population. BMC Health Service Research. 2012;12:35. 10.1186/1472-6963-12-35 PMC3305524 22333434

[33] Ware J, Kosinski M, Keller SD. A 12-item short-form health survey: Construction of scales and preliminary steps of reliability and validity. Medical care. 1996;34:220–33. 8628042 10.1097/00005650-199603000-00003

[34] Kessler RC, Andrews G, Colpe LJ, Hiripi E, Mroczek DK, Normand SL, et al Short screening scales to monitor population prevalences and trends in non-specific psychological distress. Psychol Med. 2002;32:959–76. 12214795 10.1017/s0033291702006074

[35] Centers for Disease Control and Prevention (CDC). Behavioral Risk Factor Surveillance System Survey Questionnaire. Atlanta, Georgia: U.S. Department of Health and Human Services, Centers for Disease Control and Prevention; 2007.

[36] Butt P, Beirness D, Gliksman L, Paradis C, Stockwell T. Alcohol and health in Canada: A summary of evidence and guidelines for low risk drinking Ottawa, ON: Canadian Centre on Substance Abuse; 2011 [2013 Nov. 17]. Available from: http://www.ccsa.ca/2011%20CCSA%20Documents/2011-Summary-of-Evidence-and-Guidelines-for-Low-Risk%20Drinking-en.pdf.

[37] Haggerty JL, Lévesque JF. A new measure of availability and accommodation of healthcare that is valid for rural and urban contexts Unpublished manuscript. Department of Family Medicine,McGill University(n.d).

[38] Haggerty JL, Roberge D, Freeman GK, Beaulieu C, Breton M. Validation of a generic measure of continuity of care: when patients encounter several clinicians. Annals of family medicine. 2012;10(5):443–51. 10.1370/afm.1378 22966108 PMC3438212

[39] Safran DG, Kosinski M, Tarlov AR, Rogers WH, Taira DH, Lieberman N, et al The Primary Care Assessment Survey: tests of data quality and measurement performance. Medical care. 1998;36:728–39. 9596063 10.1097/00005650-199805000-00012

[40] Stewart AL, Napoles-Springer A, Pérez-Stable E, POsner SF, Bindman AB, Pinderhughes HL, et al Interpersonal Processes of Care in diverse populations. The Milbank Quarterly. 1999;77(3):305–39. 10526547 10.1111/1468-0009.00138 PMC2751132

[41] Belzile E, Sanche S, McCusker J, Vadeboncoeur A, Ciampi A, Levesque JF. A measure of emergency department use based on administrative data Montréal, Canada: Mary's Hospital Centre; 2011. Available from: http://s3.amazonaws.com/stmary-research/attachments/000/000/048/original/11d7c5871fe5b810091286ef300ffa02.

[42] Harrell FE Jr., Shih YC. Using full probability models to compute probabilities of actual interest to decision makers. Int J Technol Assess Health Care. 2001;17(1):17–26. . 11329842 10.1017/s0266462301104034

[43] Brown H, Prescott RJ. Applied Mixed Models in Medicine, 2nd Edition New York: Wiley; 2006. 478 p.

[44] Enard KR, Ganelin DM. Reducing preventable emergency department utilization and costs by using community health workers as patient navigators. J Healthc Manag. 2013;58(6):412–27; discussion 28. 24400457 PMC4142498

[45] Boulware LE, Marinopoulos S, Phillips KA, Hwang CW, Maynor K, Merenstein D, et al Systematic review: the value of the periodic health evaluation. Annals of internal medicine. 2007;146(4):289–300. . 17310053 10.7326/0003-4819-146-4-200702200-00008

