# Supplementary material for: Personal Characteristics and Experience of Primary Care Predicting Frequent Use of Emergency Department: A Prospective Cohort Study
Source: PLoS One. 2016 Jun 14;11(6):e0157489. doi: 10.1371/journal.pone.0157489 (PMC4907452; doi:10.1371/journal.pone.0157489)
Supplement: S1 Appendix — (DOCX) [file pone.0157489.s001.docx]

**SI Appendix.** **Plausibility of the assumption that the relationship between predictor and ED use was similar for both years.**

The final model made an assumption about the impact of predictors on the outcome: the impact is independent of the year the outcome was measured. To illustrate the statement, we can imagine two groups of individuals. The first group, whose predictors are measured at baseline, is followed for a year to identify frequent ED usage. The same procedure is repeated the following year for a second group. Our assumption states that if both groups display the same characteristics at baseline, then they will exhibit the same likelihood of frequent ED usage. In the case of this study, both groups are composed of the same patients. To assess the validity of our assumption, we fitted both datasets separately: one dataset included predictors measured at T1 and outcome measured at T2, while the second included predictors measured at T2 and outcome measured at T3. We fitted each dataset using the final model. The results are reported in table 1 below.

**Table 1. Final model results stratified by outcome year.**

|  |  | **YEAR T2** | | **YEAR T3** | | **COMBINED YEARS** | |
| --- | --- | --- | --- | --- | --- | --- | --- |
| **Confounding variables** | |  |  |  |  |  |  |
|  | Age | 0.995 | [0.971;1.018] | 0.993 | [0.966;1.021] | 0.997 | [0.979;1.014] |
|  | Female gender | 0.799 | [0.472;1.351] | 1.255 | [0.651;2.418] | 1.012 | [0.676;1.514] |
|  | Lowest SES cluster | 1.424 | [0.751;2.702] | 2.758 | [1.356;5.609] | 1.897 | [1.19;3.026] |
|  | Rurality | 0.576 | [0.325;1.021] | 0.888 | [0.449;1.754] | 0.682 | [0.438;1.061] |
| **Intrinsic Patient Factors** | |  |  |  |  |  |  |
|  | Multimorbidity | 1.107 | [1.036;1.182] | 1.132 | [1.049;1.222] | 1.096 | [1.044;1.15] |
|  | Mental heath functioning | 1.01 | [0.981;1.041] | 0.996 | [0.961;1.032] | 1.004 | [0.983;1.026] |
|  | High risk alcohol consumption | 0.643 | [0.29;1.425] | 0.633 | [0.214;1.872] | 0.667 | [0.361;1.231] |
|  | High risk psychological distress | 1.978 | [0.776;5.041] | 1.46 | [0.467;4.564] | 1.661 | [0.842;3.279] |
| **Primary Care Experience** | |  |  |  |  |  |  |
|  | Organisational accessibility | 0.596 | [0.425;0.835] | 0.67 | [0.468;0.96] | 0.609 | [0.48;0.772] |
|  | Empowerment | 1.2 | [0.894;1.611] | 0.806 | [0.533;1.217] | 0.976 | [0.779;1.221] |
|  | Number of coordination issues | 1.112 | [0.963;1.285] | 1.331 | [1.113;1.591] | 1.195 | [1.075;1.328] |
|  | Interpersonal communication | 1.244 | [0.877;1.764] | 1.163 | [0.756;1.789] | 1.179 | [0.916;1.517] |
|  | Self-efficacy | 0.795 | [0.604;1.046] | 1.131 | [0.791;1.617] | 0.947 | [0.773;1.16] |
|  | Unmet health care needs | 0.878 | [0.432;1.785] | 0.921 | [0.417;2.034] | 0.906 | [0.552;1.486] |
|  | Having a Family Physician | 0.685 | [0.299;1.57] | 0.457 | [0.183;1.14] | 0.605 | [0.33;1.112] |
|  | Complete annual checkup | 0.595 | [0.318;1.113] | 0.584 | [0.259;1.316] | 0.596 | [0.374;0.95] |

No significant deviation from our assumption was noticed on the basis that all confidence intervals overlapped and contained the point estimate obtained from the combined analysis. These results should be interpreted with care. The model was too complex to fit data years separately without risking overfitting. Overfitting occurs when the model fits random noise. This impacts parameter reliability. According to general guidelines, to avoid overfitting, we could have fitted a maximum of 9 parameters using the first year of data, and 6 or 7 the second year.[Harrell (2015)] However and in both cases, 15 parameters were fit.

Similarly, we did not notice a significant deviation of predictor effects as a function of the cohort, as displayed in Table 2. Also, the model had a higher risk of overfitting the data when separating cohorts.

**Table 2. Final model results stratified by cohort.**

|  |  | **Population Cohort** | | **Clinical Cohort** | | **Combined Cohorts** | |
| --- | --- | --- | --- | --- | --- | --- | --- |
| **Confounding variables** | |  |  |  |  |  |  |
|  | Age | 1.008 | [0.984;1.031] | 0.978 | [0.948;1.009] | 0.997 | [0.979;1.014] |
|  | Female gender | 0.766 | [0.465;1.263] | 1.505 | [0.657;3.45] | 1.012 | [0.676;1.514] |
|  | Lowest SES cluster | 1.774 | [0.97;3.247] | 2.743 | [1.176;6.398] | 1.897 | [1.19;3.026] |
|  | Rurality | 0.581 | [0.34;0.992] | 1.245 | [0.508;3.053] | 0.682 | [0.438;1.061] |
| **Intrinsic Patient Factors** | |  |  |  |  |  |  |
|  | Multimorbidity | 1.104 | [1.038;1.174] | 1.109 | [1.016;1.21] | 1.096 | [1.044;1.15] |
|  | Mental heath functioning | 1.009 | [0.98;1.038] | 0.998 | [0.963;1.034] | 1.004 | [0.983;1.026] |
|  | High risk alcohol consumption | 0.595 | [0.274;1.289] | 0.672 | [0.22;2.055] | 0.667 | [0.361;1.231] |
|  | High risk psychological distress | 2.751 | [1.131;6.692] | 0.831 | [0.263;2.622] | 1.661 | [0.842;3.279] |
| **Primary Care Experience** | |  |  |  |  |  |  |
|  | Organisational accessibility | 0.735 | [0.546;0.989] | 0.415 | [0.261;0.66] | 0.609 | [0.48;0.772] |
|  | Empowerment | 0.874 | [0.656;1.164] | 1.088 | [0.723;1.637] | 0.976 | [0.779;1.221] |
|  | Number of coordination issues | 1.242 | [1.087;1.419] | 1.117 | [0.924;1.352] | 1.195 | [1.075;1.328] |
|  | Interpersonal communication | 1.13 | [0.816;1.563] | 1.305 | [0.821;2.072] | 1.179 | [0.916;1.517] |
|  | Self-efficacy | 0.953 | [0.73;1.243] | 0.993 | [0.701;1.406] | 0.947 | [0.773;1.16] |
|  | Unmet health care needs | 0.687 | [0.341;1.381] | 1.384 | [0.619;3.098] | 0.906 | [0.552;1.486] |
|  | Having a Family Physician | 0.492 | [0.244;0.994] | 1.021 | [0.245;4.252] | 0.605 | [0.33;1.112] |
|  | Complete annual checkup | 0.511 | [0.273;0.956] | 0.649 | [0.298;1.415] | 0.596 | [0.374;0.95] |
